# Supplementary material for: Analysis of cervical and breast cancer screening behavior and its influencing factors among urban and rural women in Beijing
Source: PLoS One. 2025 Jan 24;20(1):e0316898. doi: 10.1371/journal.pone.0316898 (PMC11760588; doi:10.1371/journal.pone.0316898)
Supplement: Attachment — Submitted filename: Response to Reviewers.docx [file pone.0316898.s002.docx]

Dear Editors and Reviewers:

Thank you for your letter and for the reviewers’comments. I have changed the text format as requested. The data of this study were uploaded as supporting information,which is available to all. And I have re-uploaded "S1 Database.primary data". The following is the response to the questions raised by the reviewers.

1. Could you please explain what is unique about your study? Representative due to the Capital city of China?

This study mainly discusses the screening behavior and influencing factors of women with two cancers in urban and suburban areas of Beijing. The sample size is relatively large, which represents the current situation of two cancer screening in the capital of China to some extent.

2. For the discussion section, the authors only compared the findings with several cities that are not well-known from my perspective. Please explain in more detail the clinical meaning of the comparisons.

Discussion section compares the results of the survey in Beijing and different provinces in China, can reflect the differences in different regions within China. Through comparison, the gap can be found, and the authenticity of the data can be reflected to a certain extent. In the mean time, global data are added to the article, and the gap is found through comparison at home and abroad, which provides a basis for better screening of the two cancers and promoting the implementation of the screening policy of the two cancers.

3. The results section is just a simple list of data, lacking highly refined information and conclusions.

The data are described in the results section of this paper, and the results are refined and discussed in the discussion section.

4. In table 1, what is the definition of the 'Self-rated health status'?

Self-rated health status is the subjective evaluation of one's own health. This study divides the health status into three categories: poor, general and good, which has been explained in the index definition section of the article.

5. Currently, there are four main types of basic health insurance in China. What is the kind of 'Hospitalization insurance' in this study?

Many thanks for proposing and discovering the problem. In this study, medical insurance refers to basic medical insurance, which is a social insurance system established to compensate workers for the economic losses caused by disease risk. The “Hospitalization insurance” in the table is the “Medical insurance” in the text, which has been made in the table.
